# Hidradénocarcinome du cuir chevelu : à propos d'un cas

**DOI:** 10.11604/pamj.2014.17.102.3485

**Published:** 2014-02-10

**Authors:** Ali Sbai, Asmae Ouabdelmoumene, Farid Naciri, Mohammed Elhfid, Loubna Mezouar

**Affiliations:** 1 Centre Régional d'Oncologie Hassan II, Oujda, Maroc; 2 Faculté de Médecine et de Pharmacie, Université Mohammed Premier,Oujda, Maroc

**Keywords:** Hidradénocarcinome, exérèse, radiothérapie, adjuvante, curage, prophylaxie, Hidradenocarcinoma, excision, radiotherapy, adjuvant, cleaning, prophylaxis

## Abstract

L'hidradénocarcinome est une tumeur annexielle maligne extrêmement rare. Caractérisée par la fréquence des récidives locorégionales et des métastases à distance. Le diagnostic histopathologique de malignité se fait sur des critères architecturaux et cytologiques particuliers .la chirurgie d'exérèse large représente l'essentiel du traitement. Une radiothérapie adjuvante s'avère obligatoire en cas de facteurs de récidive locale. Le curage ou la radiothérapie prophylactiques des aires ganglionnaires régionales non envahies pourraient jouer un rôle primordial dans la réduction du risque de récidives locorégionales.

## Introduction

L'hidradénocarcinome appelé aussi hidradénome malin est une tumeur annexielle maligne extrêmement rare qui représente moins de 0,001% [1, 2]. C'est une tumeur agressive qui métastase très souvent, aussi bien vers les ganglions lymphatiques régionaux que dans les viscères à distance. Elle survient préférentiellement au niveau de la tête et du cou [3], rarement au niveau des membres. Le diagnostic histopathologique de malignité se fait sur des critères architecturaux et cytologiques particuliers [4, 5]. L'excision chirurgicale large représente l'essentiel du traitement, pourtant et vu le risque élevé de récidives locales allant de 10 à 50% [6] ; une radiothérapie adjuvante s'avère nécessaire dans plusieurs cas [7].dans les formes métastatiques plusieurs protocoles de chimiothérapie ont été utilisés, la plupart à base de 5fluorouracil [8, 9]. Son pronostic reste très mauvais. Nous rapportons une observation d'un homme de 60 ans présentant un hidradénocarcinome du cuir chevelu.

## Patient et Observation

**Figure 1:**
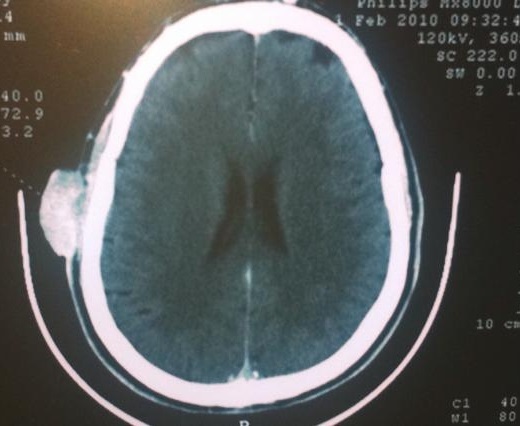
TDM du cuir chevelu montrant un processus tumoral temporal droit fortement vascularisé sans extension locale

**Figure 2:**
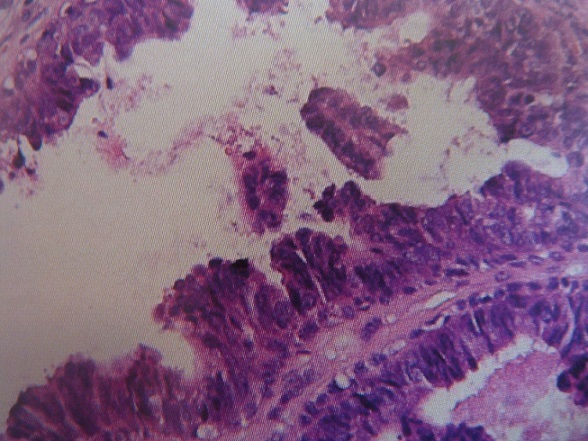
Hidradénocarcinome Coupe histologique fort grandissement x 200

Patient de 60 ans sans antécédents pathologiques particuliers ,qui rapporte la survenue depuis l´année 2009 d'un nodule du cuir chevelu localisé au niveau de la région temporale droite augmentant progressivement de taille . il n'a consulté qu'une année plus tard . Une TDM du cuir chevelu a montré un processus tumoral temporal droit fortement vascularisé sans extension locale (Figure 1). Un bilan d´extension (radiographie pulmonaire et échographie abdominale) s'est révélé normal. Il a bénéficié d'une exérèse du nodule du cuir chevelu. L´étude histologique a permis de montrer la présence d'une tumeur annexielle maligne de type hidradénocarcinome apocrine (Figure 2) d'une taille de 4 cm dont les limites d'exérèse étaient saines : à 0,8 cm de chaque côté (grand axe), à 0,5 et 0,4 cm en transversal et à 1,5 cm de la section profonde. Une reprise chirurgicale a été faite pour obtenir des limites d'au moins 1 cm. L´étude histologique a permis de montrer l'absence de résidu tumoral. Vu l'absence de facteurs de récidive locale (les emboles vasculaires, l'engainement péri nerveux, les limites envahies, la profondeur de l'infiltration et le caractère anaplasique) on a décidé une surveillance seule. Après 39 mois de bon contrôle locale et à distance, il a développé une récidive ganglionnaire latérocervicale gauche (adénopathies jugulocarotidiennes et spinales droites fixées) avec absence de métastases à distance. On opté pour une chimiothérapie néo adjuvante à base de 5 fluorouracil et de cispliatne, suivie en cas de bonne réponse d'un curage ganglionnaire cervical bilatéral et d'une radiothérapie sur les aires ganglionnaires cervicales bilatérales. Actuellement il vient de terminer sa deuxième cure de chimiothérapie avec une réduction partielle de la taille des adénopathies.

## Discussion

L'hidradénocarcinome est une tumeur annexielle maligne extrêmement rare qui représente moins de 0,001% [1, 2]. Elle a été rapportée pour la première fois en 1954 par Keasby and Hadley [10]. C'est une tumeur agressive qui métastase très souvent, aussi bien vers les ganglions lymphatiques régionaux que dans les viscères à distance. Elle survient préférentiellement au niveau de la tête et du cou [3], rarement au niveau des membres. Le diagnostic de certitude est basé sur l'étude anatomopathologique qui montre une prolifération tumorale intradermique en nappe, comportant une différenciation sudorale et découpée par un stroma dense et hyalinisé avec pléomorphisme nucléaire et figures de mitoses[4, 5]. La chirurgie basée sur une exérèse large avec des limites saines est le gold standard [7, 11, 12]. Le rôle du curage ganglionnaire régional est controversé dans la littérature mais la plupart des auteurs s'accordent ;en cas d'absence de métastase à distance ; sur la nécessité de la dissection et l'irradiation combinées des ganglions envahis alors que les ganglions non envahis doivent être soit disséqués soit irradiés [7, 11, 12]. Une radiothérapie adjuvante s'avère nécessaire en cas de présence de facteurs de récidive locale : (les emboles vasculaires, l'engainement périnerveux, les limites envahies, la profondeur de l'infiltration et le caractère anaplasique)[7, 13]. Dans les formes métastatiques plusieurs protocoles de chimiothérapie ont été utilisés, la plupart à base de 5 fluorouracil [8, 9]. Chez notre patient et en raison de l'absence de facteurs de récidive locale, une chirurgie large a fait l'essentiel du traitement . Cependant la récidive ganglionnaire cervicale après un intervalle libre de 39 mois nous a interpellé sur le rôle qu'auraient pu jouer un curage ou bien une radiothérapie prophylactiques des aires ganglionnaires cervicales.

## Conclusion

L'hidradénocarcinome est une tumeur très agressive caractérisée par la fréquence des récidives locorégionales et des métastases à distance. La chirurgie d'exérèse large représente l'essentiel du traitement. Une radiothérapie adjuvante s'avère obligatoire en cas de facteurs de récidive locale. L'expérience de notre patient conforte la place du curage ou de la radiothérapie prophylactiques des aires ganglionnaires régionales non envahies.
